# Plasmid-encoded colistin resistance: *mcr*-one, two, three and counting

**DOI:** 10.2807/1560-7917.ES.2017.22.31.30588

**Published:** 2017-08-03

**Authors:** Jan Kluytmans

**Affiliations:** 1 Amphia Hospital Breda and University Medical Centre Utrecht

**Keywords:** mcr-1, mcr-2, mcr-3, ESBL, Enterobacteriaceae, Escherichia coli

In November 2015, the first description of plasmid-mediated colistin resistance (*mcr-1* gene) was reported from China in food animals, food and humans [1]. Many reports from all over the world have followed since. The reported rates vary considerably, ranging from sporadic findings up to 67% in *Escherichia coli* isolates fromTunisian chicken [2]. However, the rates have been consistently higher in livestock than in humans. This points to a reservoir in animals with spill over to humans. Until recently, colistin use in humans has been limited but it has been used extensively in veterinary medicine for decades, both as curative treatment and for prevention of disease [3]. The amount of use in livestock varies enormously. In Europe, for example, in 2013, the annual colistin sales in some countries exceeded 20 mg per population corrected unit (PCU) while in other countries the sales were below 1 mg/PCU. Following the detection of *mcr-1*, the European Medicines Agency updated their advice on the use of colistin in humans and animals [3] with the aim of reducing the use in animals by 65% in the coming years. Quantitative targets of 5 mg/PCU and 1 mg/PCU have been set for a reduction in high and medium consuming countries, respectively.

In the summer of 2016, a group from Belgium reported a new variant of the plasmid-mediated colistin resistance gene, *mcr-2* [3]. Xavier et al. studied colistin-resistant *E. coli* strains from pigs and calves and found *mcr-2* more frequently than *mcr-1*. In this issue of *Eurosurveillance*, there are three reports on a third variant, *mcr-3*. In one of the studies, *mcr-3* was detected in an *E. coli* isolate from a patient with a bloodstream infection who had recently visited Thailand [4]. The travel history in combination with the extended-spectrum beta-lactamase (ESBL) marker in the same strain (CTX-M55) strongly suggests that the patient acquired this multi-resistant *E. coli* in Asia. Travel outside of Europe is known to be a risk factor for acquisition of resistant bacteria. In a recent study, healthy travellers to South East Asia had a risk of acquisition of ESBL-producing *Enterobacteriaceae* as high as 75% [5]. In another study, the *mcr-1* gene was detected in stool from 5% of travellers to Asia or South Africa upon their return to the Netherlands [6]. Although data from Asia are scarce, there are multiple indicators that resistance to many antibiotics, including colistin, is more prevalent in this continent than in Europe. Structural and sustained surveillance of antimicrobial resistance in Asia is limited, with scarce data from humans and practically no data from livestock and the environment. In contrast, in Europe, surveillance has become more standardised and in some countries it has become exemplary, as illustrated by the *mcr-3* findings in this issue. Denmark has started a national surveillance of ESBL and carbapenemase-producing strains. All resistant strains causing invasive infections are systematically analysed by Whole Genome Sequencing (WGS). This approach enables the researchers to search for the presence of new genes, like *mcr-3*, once they have been identified. The patient infected with an *E. coli* strain carrying the *mcr-3* gene was identified by searching this database [7]. Also, WGS data enable comparisons between countries with an unprecedented granularity. The Danish strategy to perform WGS on resistant strains is an example, how microbiological surveillance ideally should be performed in this era of antimicrobial resistance.

Further *mcr-3* findings, in another pathogen, are also reported from Denmark. Litrup et al. et al. identified 10 human cases in samples of ca 2,500 *Salmonella* spp. from a collection spanning from 2009 to 2017 [8]. Cases were spread over the entire period without a clear trend over time. Interestingly, eight of 10 isolates harboured CTX-M-55 in addition to *mcr-3*. Five of the concerned individuals reported recent travel to Thailand or Vietnam, for three there was no information on travel and two had no travel history. From this report the relation with travel to Asia is once again obvious as is the linkage with the ESBL variant, which was also present in the human case with *E. coli*, CTX-M-55. However, at least two cases had no travel history and this points towards acquisition within Denmark. The authors discuss food as a possible source, which seems plausible considering that *Salmonella* infections are often caused by contaminated food items. However, other sources cannot be excluded.

The third report concerns an *E. coli* strain of bovine origin in Spain [9]. This finding was part of a surveillance project for ESBL-producing bacteria in food production animals during 2015. Among 152 ESBL producers, six were colistin-resistant and in five of them the *mcr-1* gene was detected. One strain harboured both *mcr-1* and *mcr-3*. All colistin-resistant strains were resistant to many classes of antibiotics that are considered of critical importance for humans. The strain with the two *mcr* variants was only susceptible to carbapenems and tigecyclin.

## What should the conclusions from these findings be?

First, antimicrobial resistance is growing, both quantitatively i.e. incidence of resistant strains, and qualitatively i.e. diversity of the underlying mechanisms. Within 2 years after the first report of plasmid-encoded colistin resistance, three variants of the gene have been identified. It should be noted that this is not only due to the recent emergence of new variants but also due to the recent identification of these variants by the use of sophisticated tools that have become available. In this case, the first isolate with *mcr-3* was retrospectively detected in an isolate from 2009, the first year covered by the surveillance project.

Second, surveillance is becoming more powerful. The broad application of WGS is a good example of a new technique with unprecedented granularity combined with a high level of standardisation. The costs of this technique are getting lower and lower, making it an attractive method for broader implementation. Improved surveillance is especially important in areas where antimicrobial resistance is high and surveillance is not structurally performed, such as in parts of Asia and Africa.

Third, the use of antibiotics is known to be the main driver for antibiotic resistance [10]. Colistin is now considered to be of critical importance for humans, which has urged a reconsideration of the use in livestock [3]. European countries show large and unexplained differences in the amount of use of antibiotics in general and colistin in livestock in particular. This strongly suggests that high-consuming countries should be able to reduce the amount used substantially. In the global context, the lack of information on the use of antibiotics and the presence of resistance in parts of areas outside of Europe like Asia, Africa and the Americas is cause of concern. As a positive sign it should be mentioned that China has taken a first step towards limiting development of antimicrobial resistance by banning the use of colistin as a feed additive [11].

Finally, although the emergence of resistance to colistin is worrying, the prevalence in humans is still low today. In the report in this issue, almost 1,200 recently collected strains of ESBL- and carbapenemase-producing *Enterobacteriaceae* were screened and one case of *mcr-3* was found [7]. Apparently, the spillover from the animal reservoir to humans in Denmark is limited at this time. The situation in Denmark may not be representative for other settings. For example, a paper from Rosslini et al, in this issue [ref 12] shows that resistance in Italy is high. Their surveillance project (in October 2013) in several Italian hospitals, found that a quarter of all *Klebsiellapneumoniae* isolates from hospitalised patients were resistant to carbapenems. From these isolates almost 40% were not susceptible to colistin. It is unclear if this was caused by *mcr-1* as no further molecular characterisation was performed. The rising levels of resistance around the globe warrant improved surveillance efforts in humans, animals and the environment. This is of most importance in areas where antimicrobial resistance can be expected to be high. This may be a challenge but it can be rewarding: there are good chances to find *mcr-4, 5, 6* and so on for those who start looking for it.

## Editorial note added in proof:

After this editorial was prepared, we received an additional article describing the discovery of an *mcr*-4 gene in Europe. This article is now also published in this issue.
